# Measuring Electromagnetic Field Activity Generated by Neurons In Vivo by Humans With Thoughts of Repetitive Motor Activities and Emotional Thoughts

**DOI:** 10.7759/cureus.23332

**Published:** 2022-03-20

**Authors:** James Wiginton, James Brazdzionis, Tye Patchana, Paras Savla, James Hung, Yongming Zhang, Dan E Miulli

**Affiliations:** 1 Neurosurgery, Riverside University Health System Medical Center, Moreno Valley, USA; 2 Electrical Engineering, Quasar Federal Systems, San Diego, USA; 3 Medical Physics, Quasar Federal Systems, San Diego, USA; 4 Neurosurgery, Arrowhead Regional Medical Center, Colton, USA

**Keywords:** development and assessment, medical device technology, helmet, thought, neurons, electromagnetic field, new technologies in neurosurgery

## Abstract

Background

In this study, a novel method of electromagnetic field (EMF) measurements of the human brain has been performed to evaluate neuronal activity. This measurement in a non-contact, non-invasive, continuous manner through the human skull and scalp in the standard environment is completed through a lightweight inexpensive helmet. We sought to further delineate whether specific activities of complex thought can be identified using this non-invasive technique.

Methodology

Non-clinical human subject volunteers donned a lightweight helmet with attached sensors and performed activities of motor movement, specific motor imagery, and specific emotional imagery synchronized to an audible tone for consistency. The human subjects’ brain EMF was recorded and analyzed.

Results

The novel method of continuously recording real-time human brain EMF was able to determine differing brain activity between individuals performing motor movement, motor imagery, and emotional imagery in a non-contact manner at different distances from the scalp.

Conclusions

It may be possible to measure specific human brain activity using EMF in a non-invasive fashion. Emotional imagery, motor imagery, and motor movement generate different EMFs that have different discernible forms compared to baseline activity.

## Introduction

Non-invasive neuroimaging modalities have advanced throughout recent decades. These advances in imaging technologies and new applications of functional magnetic resonance imaging and diffusion tensor imaging have led to renewed interest in developing real-time functional imaging.

The central nervous system is functionally an electrochemical circuit with resultant charge flowing to and from neurons. This current subsequently generates magnetic fields. As previously documented in Wiginton et al., this electromagnetic field (EMF) can be measured in a non-invasive fashion in humans [1]. Further, it has been documented that repetitive motor activities can be measured in a similar manner [2]. More complex thoughts such as emotion and motor planning are yet to be investigated. To build upon prior research we sought to further delineate whether specific activities of complex thought can be identified using this previously documented non-invasive fashion. In addition, we hoped to identify if there were specific patterns that could be identified within a single volunteer or among a group of volunteers to create waveform maps of differing activities.

## Materials and methods

Institutional Review Board approval was obtained from Arrowhead Regional Medical Center entitled “In-vivo non-contact remote measurement of neuronal activity” protocol #21-05. A lightweight helmet and four proprietary EMF passive sensors (BS-1000; Quasar Federal Systems, San Diego, CA) were configured to bi-temporal and bi-frontal orientation in a non-contact manner [2]. The sensors were labeled as Bx, By, Bz, and B319. The EMF was recorded from two volunteer human subjects during different activities and thoughts. The data were analyzed for patterns of predictability.

All tests were performed with a background metronome (non-ferromagnetic) placed outside of the Faraday shield which produced sound at 120 bpm (2 Hz). This allowed the detection of a reproducible recording of brain activity from subjects whether they were tapping (motor movement) or had thoughts of tapping (motor imagery) at 2 Hz. Most tests recorded motor movement and motor imagery from the subjects’ right hand. Others tested EMF with the motor movement of both hands at 2 Hz. If motor activity speeds varied, the metronome was reset to the designated Hz value. The tapping (motor movement) was as previously described. The hand was lifted as one unit while the wrist remained on the ground, and the tips of the extended fingers were elevated 6 cm [2].

The subjects were tested inside a Faraday shield without a separate Mu-metal cage. A light-weight helmet was constructed containing two distinct Mu-metal (MuMETAL®, Magnetic Shield Corporation, Bensenville, IL) layers with an inner and outer layer of interlaced copper mesh. Sensors were each wrapped with Mu-metal foil. The sensors were placed 4.5 cm above the scalp oriented with the positive (+) side facing toward the subject’s scalp. Sensor positions were designated as the following: Bz was located at approximately the right frontal motor strip and aimed at the left motor strip; By was located approximately at the location of the right temporal lobe and oriented toward the left motor strip; B319 was located in the left frontal region and oriented toward the ipsilateral left motor strip; and Bx approximated the left temporal lobe and was aimed toward the left motor strip.

As previously described, the passive magnetic induction sensors were configured in accordance with a previous protocol [2]. They were connected to amplifiers outside the Mu-metal enclosure of the helmet. EMF signals were captured at 5,000 Hz at a distance from the Faraday shield using a laptop computer with a 16-bit National Instruments Data Acquisition Card and LabVIEW software (National Instruments Corporation, Austin, TX) and subsequently post-processed using Igor® Pro 8 software (Wavemetrics Inc., Lake Oswego, OR). The collected data were transformed using Fast Fourier transform (FFT), plotting the cumulative composite values of the voltage recorded in 20-second bins versus frequency recorded over 120-second blocks. Data were binned into 0.3 Hz from 1 Hz to 2 kHz for every 20 seconds. Waveform analysis of the FFT waves was plotted for comparison and analysis.

Each sensor was aligned intersecting the same targeted source of the left motor strip to measure the same signal of the left motor strip from different angles. The intervening brain tissue would be different between the motor strip and the corresponding sensor due to differing traversing cortical activities and neuronal tissues. Subjects were informed of testing parameters and trained in the planned activities of tapping (motor movement), thought of tapping (motor imagery), and complex thought (emotional imagery) before obtaining measurements. Each experiment was designed such that they would begin with 30 seconds of inactivity before starting the activity. The designated activity was performed for 60 seconds at predetermined frequencies determined by the metronome at 2 Hz, 3 Hz, and 3.467 Hz. Waveforms were obtained during all phases of the testing.

All motor imagery tasks initially involved 30 seconds of rest and meditative techniques interspersed with 60 seconds of the subject thinking of tapping the extremity without an actual physical movement of the extremity. The subject was observed to ensure no movement occurred. The order of motor imagery interspersed with 30 seconds of rest were as follows: right hand, left hand, right foot, left foot, right side (combination of right hand and right foot), left side (combination of left hand and left foot), and whole body (combination of right and left hands and right and left feet).

For emotional imagery testing, the sensor configuration was changed. Two sensors were placed perpendicular to the temporal lobe 4.5 cm from the scalp and two sensors were placed perpendicular to the prefrontal cortex 4.5 cm from the scalp using anatomic landmarks. The emotional imagery task involved the subject invoking a total of five emotion-generating thoughts with no physical movement. The tests started with 30 seconds of inactivity and meditation followed by 60 seconds of a single emotion-generating thought. This was repeated for each of the five thoughts. The first four emotion-generating visualizations consisted of imagining the subject’s family member in a specific circumstance repeated every half second (2 Hz). The fifth emotion-generating thought consisted of imagining a different person in a specific circumstance repeated every half second (2 Hz).

Tests 1-7

A synchronizing stimulus protocol was utilized to allow for detecting reproducible brain activity from the subjects. For tests 1-5, the sensors were positioned positive (+) toward the scalp. Right sensors were Bx (front) and B319 (posterior). Left sensors were Bz (front) and By (posterior). For tests 6 and 7, Bx was moved to the right temporal region, and Bz was moved to the left temporal region. The sensors were universally positioned 4.5 cm away from the skin. There was consistency in the design of each experiment: 30 seconds of rest inactivity at the start and end of each test as well as 30 seconds of rest inactivity between recording brain activity, as described above. A brief description of each test is presented in Table 1.

**Table 1:** Brief descriptions of tests 1-7.

Test	Description	Total time (seconds)
1	Background recording with intermittent 60 seconds of slight head bobbing	240
2	30 seconds of inactivity with 60-second series of thoughts about limb movement	660
3	Inactivity test for adjustment of sensitivity for proceeding tests	30
4	30 seconds of inactivity followed by thought regarding left-hand movement followed by 30 seconds of rest	120
5	Repeat of test 2 except with change in sensitivity of recording, as determined in test 3	660
6	Using new temporal position of sensors, emotion-generating, thinking-only experiment, as outlined in test 5	410
7	Repeat of emotion-generating, thinking-only experiment with unknown repeat of thought	210

Test 2 was the first attempt at measurement of human thought from the volunteer subject. This involved 30-second rest/inactivity periods interspersed with the subject thinking of tapping extremities. The order of motor imagery was as follows: right hand, left hand, right foot, left foot, right side (right hand and right foot), left side (left hand and left foot), and the entire body (right and left hands and feet). The subject performed meditative techniques during rest/inactivity periods and then concentrated thinking on tapping (without actual motor movement). The subject’s lack of movement was verified by an observer and an attached gyroscope.

Test 3 allowed the investigators to ascertain the ideal millivolt (mV) range and sensitivity gain to have maximum sensitivity in an attempt to record the subject’s thought. The typical range for previous movement testing was from 1 mV to 1 V. Test 3 revealed an ideal sensitivity range from 5 mV to 200 mV by the operator and author JH.

Given the adjustment in sensitivity as described in test 3, a repeat motor imagery experiment was performed for test 4. For this, 30 seconds of rest inactivity was followed by 60 seconds of thinking of left-hand movement followed by 30 seconds of rest/inactivity. After this, a repeat of the test 2 protocol was performed and recorded as test 5 except with this change in sensitivity obtained from test 3.

Tests 6 and 7 required reconfiguration of the sensors within the helmet. Two sensors were placed perpendicular to the temporal lobe 4.5 cm from the scalp, and two sensors were placed perpendicular to the prefrontal cortex 4.5 cm from the scalp. Because previous tests 2, 4, and 5 determined that specific brain activity could be reliably measured when thinking of tapping, it needed to be determined if the measured EMF was due to thinking of movement or micro-movement. Test 6 involved the subject thinking and visualizing five different scenes to generate emotions with no movement (emotional imagery). During this, 30 seconds of rest inactivity periods involved meditation concentration techniques, as described above. Test 7 started with a period of 30 seconds of inactivity followed by one of the emotion-generating visualization scenes. This emotion-generating visualization was a repeat of one of the five tests previously mentioned in test 6 above. After another period of inactivity, a repeat of the same emotion-generating visualization was performed. The first four emotion-generating visualizations consisted of imagining a different family member in a specific circumstance repeated every half second (2 Hz). The fifth emotion-generating visualization consisted of imagining a different person in a specific circumstance repeated every half second, 2 Hz.

Tests 8-22

Given the results from tests 1-7, it needed to be determined if the measured EMF was due to thinking of movement or micro-movement that occurred while thinking. Tests 8-22 introduced variation in Hz for tapping (tests 8-10) and thoughts of tapping (11-13). Tests 14 through 22 were performed to compare between subjects. A full explanation of each test is presented in Table 2.

**Table 2:** Brief descriptions of tests 8-22.

Test	Description	Total time (seconds)
8	30 seconds of inactivity with 60 seconds of tapping 2 Hz subject 2. No rest at end. 4.5 cm	180
9	30 seconds of inactivity with 60 seconds of tapping 3 Hz subject 2, 4.5 cm	210
10	30 seconds of inactivity with 60 seconds of tapping 3.467 Hz subject 2, 4.5 cm	210
11	30 seconds of inactivity with 60 seconds of thinking of tapping 2 Hz subject 2, 4.5 cm	210
12	30 seconds of inactivity with 60 seconds of thinking of tapping 3 Hz subject 2, 4.5 cm	210
13	30 seconds of inactivity with 60 seconds of thinking of tapping 3.467 Hz subject 2, 4.5 cm	210
14	30 seconds of inactivity with 60 seconds of tapping 2 Hz subject 1, 4.5 cm	210
15	30 seconds of inactivity with 60 seconds of tapping 3 Hz subject 1, 4.5 cm	210
16	30 seconds of inactivity with 60 seconds of tapping 3.467 Hz subject 1, 4.5 cm	210
17	30 seconds of inactivity with 60 seconds of thinking of tapping 2 Hz subject 1, 4.5 cm	210
18	30 seconds of inactivity with 60 seconds of thinking of tapping 3 Hz subject 1, 4.5 cm	210
19	30 seconds of inactivity with 60 seconds of thinking of tapping 3.467 Hz subject 1, 4.5 cm	210
20	30 seconds of inactivity with 60 seconds of tapping 2 Hz, 30 seconds of rest, 60 seconds of thinking of tapping 2 Hz, subject 2, 1 cm	210
21	30 seconds of inactivity with 60 seconds of happy thought 2 Hz, 30 seconds of rest, 60 seconds of bad thought 2 Hz, 60 seconds of happy thought 2 Hz, 30 seconds of rest, 60 seconds of bad thought 2 Hz, subject 2, 1 cm	390
22	30 seconds of inactivity with 60 seconds of left-hand tapping 2 Hz, 30 seconds of rest, 60 seconds of thinking of left-hand tapping 2 Hz, subject 2, 1 cm	210

## Results

**Figure 1:**
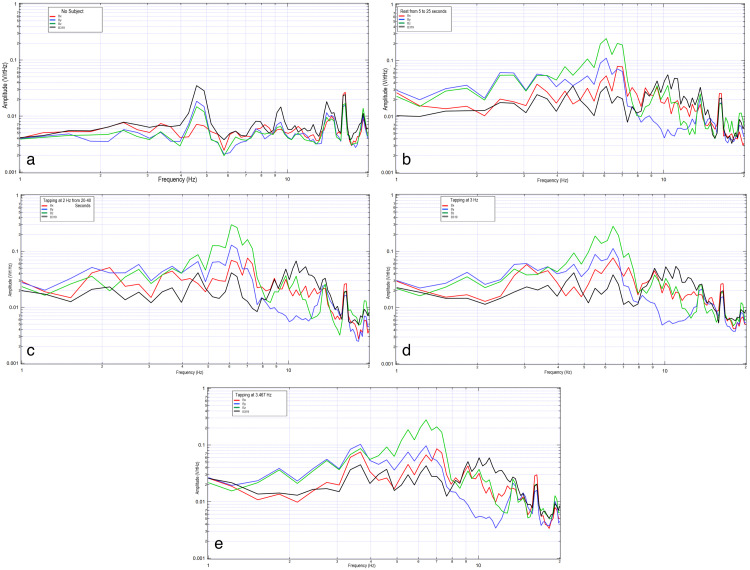
Comparison of the background without subject, subject at rest, and subject tapping at various frequencies. 1a shows background data, 1b shows subject at rest from 5 to 25 seconds, 1c shows subject tapping at 2 Hz, 1d shows subject tapping at 3 Hz, and 1e shows tapping at 3.467 Hz. In each graph, the vertical axis is sensor outputs in the frequency domain (0.001-1 V/rtHz), and the horizontal axis is the frequency (1 to 20 Hz), both in the log scale. The red curve is sensor Bx, the blue curve is sensor By, the green curve is sensor Bz, and the black curve is sensor B319.

**Figure 2:**
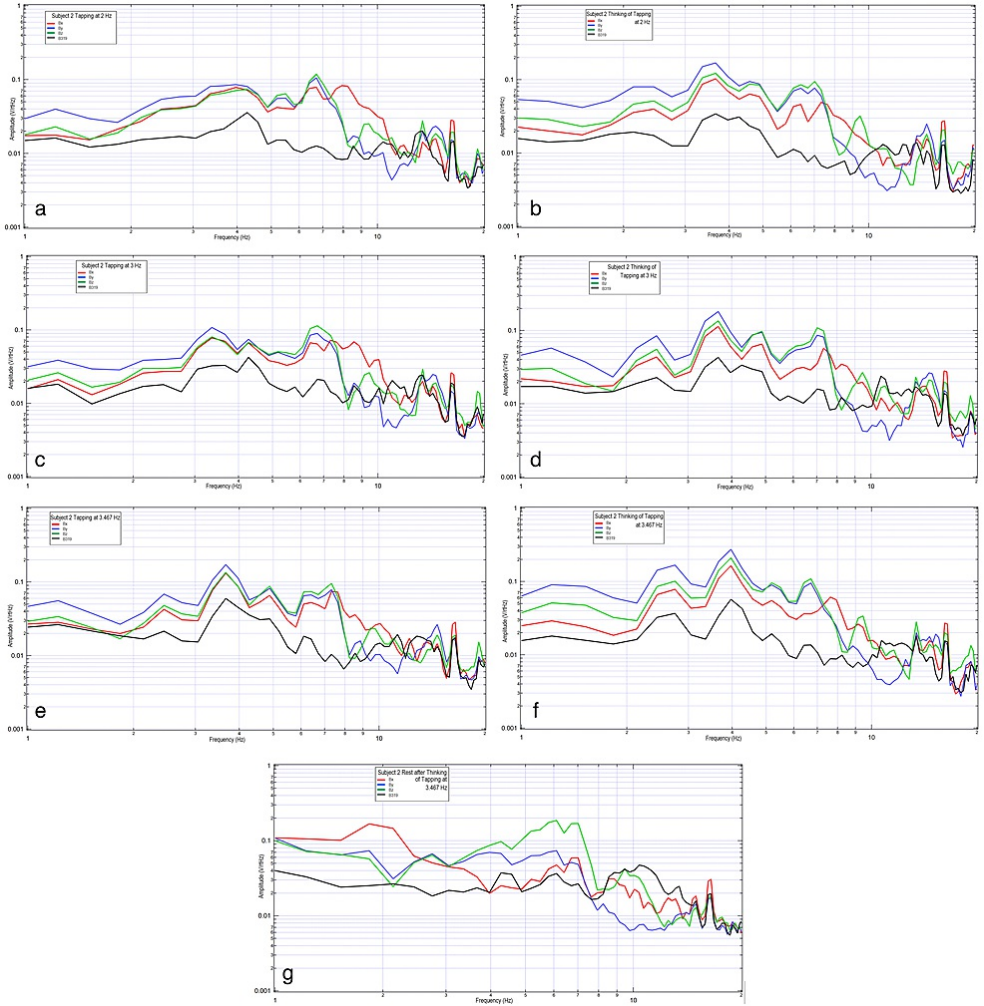
Comparison of a subject tapping versus thinking of tapping at 2Hz, 3Hz, and 3.467 Hz. 2a shows subject tapping at 2 Hz, 2b shows subject thinking of tapping at 2 Hz, 2c shows subject tapping at 3 Hz, 2d shows thinking of tapping at 3 Hz, 2e shows tapping at 3.467 Hz, 2f shows thinking of tapping at 3.467 Hz, and 2g shows subject at rest after thinking of tapping at 3.467 Hz. In each graph, the vertical axis is sensor outputs in the frequency domain (0.001-1 V/rtHz), and the horizontal axis is the frequency (1 to 20 Hz), both in the log scale. The red curve is sensor Bx, the blue curve is sensor By, the green curve is sensor Bz, and the black curve is sensor B319.

Two subjects were evaluated wearing the helmet with activities measured at rest, during right-hand motor movement at 2 Hz, 3 Hz, and 3.467 Hz, as well as when participating in motor imagery with the thought of right-hand tapping at the same frequencies. The results are shown in Figure 1 and Figure 2.

There is an identifiable inflection point for each subject during motor activities. There appears to be altered morphology between rest and motor imagery with similarities in the inflection peak when comparing motor imagery to motor movement. When comparing subjects, it appears the EMF response differed between them. Morphologically, the waves demonstrate the sharpest regions of the inflection when participating in motor movement while adopting a more rounded appearance with motor imagery. The most rounded appearance occurs at rest.

Further, the sharp shape of the graph appears dependent upon concentration. As concentration lingers, the graphic representation of the broadcasted EMF takes a more rounded shape, as seen in Figure 2g. The inflection point is seen to move in this subject to 6.5 Hz.

**Figure 3:**
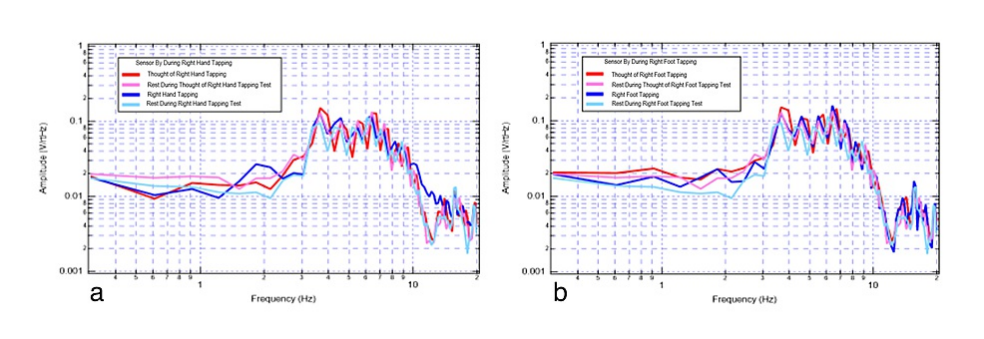
Left motor strip sensor By recording rest, tapping, and thought of tapping. 3a shows right-hand movements; 3b shows right-foot movements. Red denotes thought of right-hand tapping, pink denotes rest during thought of right-hand tapping, dark blue denotes right-hand tapping, and teal denotes rest during right-hand tapping.

Individual sensor data were extracted and plotted during these trials to evaluate changes simultaneously. In Figure 3, data from sensor By was plotted during activities of the right hand (Figure 3a) and right foot (Figure 3b) motor movement, right hand, and right foot motor imagery, and during rest periods.

**Figure 4:**
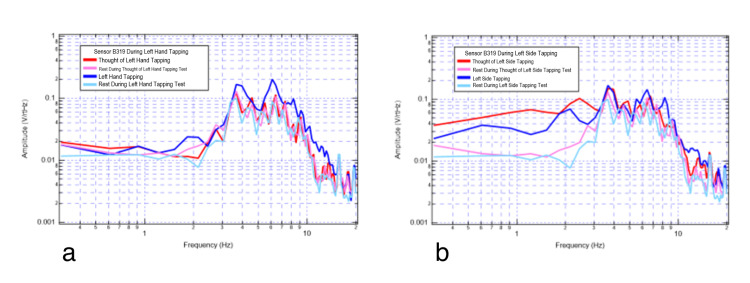
Right motor strip sensor B319 recording rest, tapping, and thought of tapping. 4a shows left-hand movements; 4b shows left-side movements. Red denotes thought of left-hand tapping, pink denotes rest during thought of left-hand tapping, dark blue denotes left-hand tapping, and teal denotes rest during left-hand tapping.

These results from different 2 Hz tests in the same individual demonstrate amplitude and shape change from rest (light blue) to the right-hand motor movement (dark blue). There are differing peaks signifying larger coordination of brain activity. There is also a change from rest (pink) to right-hand motor imagery (red), as demonstrated between 3 and 4 Hz, where the motor imagery generated a plateau-shaped wave. There are similar changes with right-foot motor movement. Similar changes were also seen in the premotor sensor (B319), as seen in Figure 4.

Sensor B319 (premotor) identified additional morphologic changes when evaluating motor imagery, rest, and motor movement. There were higher amplitude peaks during motor movement exercises compared to motor imagery. When comparing rest and motor imagery, the thought of activity demonstrated some increased plateau-like waves between 2 and 3 Hz and between 4 and 5 Hz. The largest degrees of overlap seen in Figure 3 and Figure 4 were between 3 and 4 Hz where there were overall similarities within each measured signal morphology and amplitude.

There were demonstrable changes in shapes, peaks, valleys, and amplitudes at various frequencies from rest (light blue) to the left-hand motor movement (dark blue) and rest (pink) to motor imagery of left-hand tapping (red) at 2 Hz in Figure 4a. There are similar changes with left-sided activity in Figure 4b.

**Figure 5:**
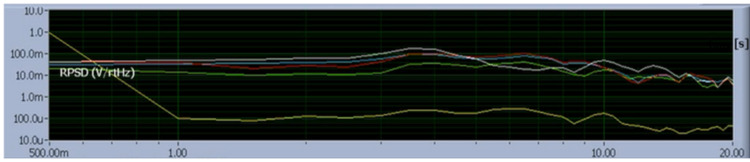
Real-time recording (screenshot) of rest without thinking about moving limbs. Demonstrating 20-second epochs of accumulated voltage/rtHz at frequencies ranging from 1 to 20 Hz. The red line is Bx, the green line is By, the black line is Bz, and the white line is B319. The yellow lower line is the gyroscope to record movement.

A gyroscope was attached to the helmet to evaluate for changes in micromovement and evaluate for the presence of motion-generated activity. These results, as seen in Figure 5, represent the real-time recording of sensor outputs in the frequency domain (V/rtHz) versus frequency (0.5 to 20 Hz).

There were demonstrable amplitudes changes identified in sensors Bx, By, Bz, and B319.

**Figure 6:**
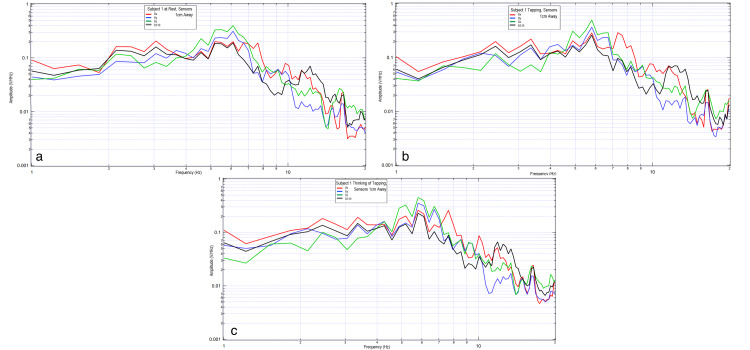
Comparison of brain EMF broadcasted 1 cm away from subject 2. Subject at rest (6a), tapping at 2 Hz (6b), and motor imagery at 2 Hz (6c). EMF: electromagnetic field

For additional evaluation, the sensors were moved closer to the subject. Sensors were placed 1 cm away from the subject while continuing to avoid the hair or scalp. The subject again participated in right-hand tapping at 2 Hz, motor imagery of right-hand tapping at 2 Hz, and interspersed rest phases between trials, as described above. The results are shown in Figure 6.

The changes between motor movement, rest, and motor imagery of tapping are better elucidated at 2 Hz. There are distinct morphology changes identified in motor imagery with plateau-shaped waves compared to motor movement and rest. Motor movement also distinctly demonstrates sharper waves than rest-based activities.

**Figure 7:**
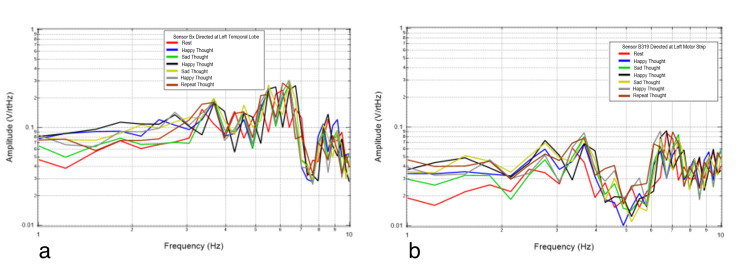
Left temporal Bx (7a) and motor strip B319 (7b) recordings from the thinking experiment. Red is rest, dark blue is happy thought, green is sad thought, black is happy thought, yellow is sad thought, gray is happy thought, and brown is repeat thought.

**Figure 8:**
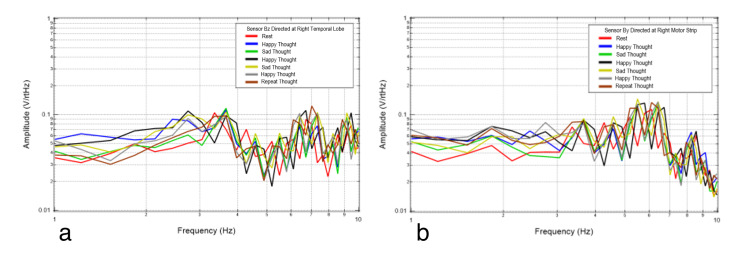
Right temporal Bz (8a) and motor strip By (8b) recordings from the thinking experiment. Red is rest, dark blue is happy thought, green is sad thought, black is happy thought, yellow is sad thought, gray is happy thought, and brown is repeat thought.

Next, emotional mental imagery was evaluated. The subject was asked to think of a happy mental image, a sad mental image, a happy mental image, a sad mental image, a happy mental image, and then to repeat one of the mental images. This was done to evaluate any differences between limbically generated signals. The repeated thought was to evaluate whether similar areas would be activated and if the repeated thought would generate a reproducible signal. The results of these trials are plotted in Figure 7 and Figure 8.

Figure 7 and Figure 8 show various mental imagery results as plotted in relation to rest (red). Mental imagery appears to be measured at a different voltage than motor activity. The repeat thought (brown) does not have an exact resemblance to any of the previous thoughts, and all the thoughts are not significantly different. The repeat thought has similarity to a previous thought at a certain frequency range and to another at a different frequency range. More specifically, the repeat thought and thought five share inflection points and similar directions of slopes. Each thought is different from that of the rest. The consistent frequencies of concentration during rest were at 3.4, 4.3, 5.2, 6.1, and 6.6 Hz. Rest is once again depicted as sharper waves.

**Figure 9:**
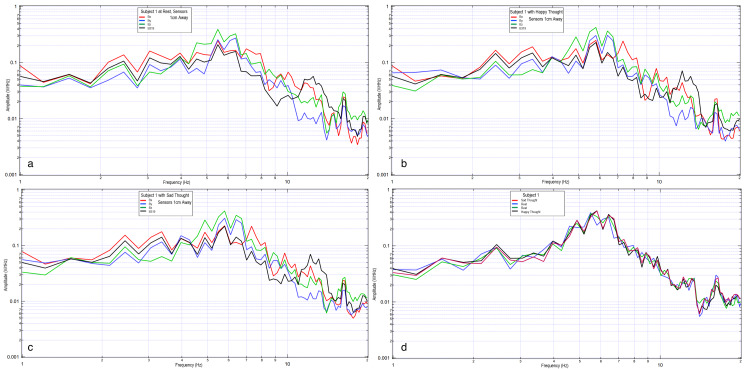
Comparison of brain activity EMF recorded from subject 2 with four sensors. Subject at rest (9a), thinking of a happy thought (9b), and thinking of a sad thought (9c). The last graph (9d) is the summary of Bz sensor during two rest periods (dark blue and green) and a happy (gray) and sad (red) thought. Sensor Bz is depicted as green and blue for rest, black for happy, and red for sad. EMF: electromagnetic field

Sensors were again moved more proximally toward the subject with sensors placed 1 cm away within the helmet construct. The subject was asked to consider a happy mental image and a sad mental image with a rest phase between. This is shown in Figure 9.

The signal once again changes shape, peaks, and polarity when the emotional imagery of happy or sad is contrasted with rest. Happy (black) and sad (red) have only minimal changes of slope polarity around 2.8 Hz, 4.6 Hz, and 7.8 Hz.

## Discussion

Normal cortical functions of the brain are the balance of normal electrochemical transduction cascades generated from neurons involved in the underlying processes [3]. The balance of these cascades leads to larger-scale depolarizations that result in our conscious thoughts, movements, emotions, and activities. These depolarizations result in changes in polarization and thus an electrical current generates an EMF [3]. Therefore, the generation of these EMF signals should functionally be localized to the specific areas during activity referable to the activity itself. Previous studies by Wiginton et al. and Brazdzionis et al. identified that sensors can be utilized to measure cortical activity in a non-contact manner and that cortically generated EMF from motor-based activities can be measured [1,2]. The current investigation was an attempt to elucidate whether motor imagery or emotional imagery would generate an EMF large enough to be measured by these sensors. The results show that these signals were indeed captured in a real-time, continuous, non-invasive, non-contact manner. This was completed using a small, light-weight, shielded helmet with real-time magnetic field sensors. The amplitude activity was the sum of repeated measurements within an identified bin using FTT, and therefore, may be a measurement of the frequency of a set of neurons firing generating the same EMF.

When evaluating the EMF generated by the subject during rest, motor movement, and motor imagery, there was a similar location of measured EMF from 2.0 to 12.5 Hz in regards to the location of the peaks and valleys (Figure 6). There were further morphologic differences comparing these activities to rest. Overall, motor movement and motor imagery grossly appeared the most similar which may indicate that similar cortical-based activity was measured between the two activities despite active motor firing. The differences between resting and motor movement and imagery indicate that there may be a difference in measured activity correlating to the generated brain EMF.

It is theorized that neuronal sources generating the dominant magnetic field at a specific frequency may be identified by multiple sensors. This measured EMF from an individual for the same activity with the same configuration of sensors reproducibly should yield peaks and valleys in a similar shape. Our results identify similar rest activities among trials. Further similarities were identified and reproducible in the activities of rest, motor movement, and motor imagery in the same subject.

When emotional imagery is passively recorded from non-contact EMF sensors, those thoughts appear to broadcast an EMF differing from rest activity, as seen in Figure 7 and Figure 8. These were demonstrably different in morphology than rest-phase EMF data. The EMF identified appeared to have unique characteristics compared to motor imagery. Therefore, different unique activities appear to generate unique EMF signal characteristics which may be referrable to the active cortical region of interest generating the signal. Although emotional imagery appeared to generate a unique EMF compared to rest and motor activities, the reproducibility of the generated EMF for differing emotional imagery was limited as there were differences in the measured amplitudes despite repeated emotional imagery. This may be due to subtle differences in the emotion and intensity of emotion generated by the individual.

EMF sensors with a maintained orientation record the same shape for a specific activity. This is demonstrated by results with equivalent cumulative voltage peaks and troughs at various frequencies. The more areas active at a specific frequency the stronger the signal voltage and the greater the voltage amplitude if the sensor angle is appropriately aligned. In the evaluation of our results, the region between approximately 5 and 7 Hz seemed to have the highest overall amplitudes and may be an area of interest for future investigation of cortically generated EMF.

Thought of a motor action through motor imagery has been found to result in the motor cortex being similarly stimulated as when the actual motor activity occurs [4]. Thus, the sensor most stimulated depends on the orientation of the sensor to the EMF of the corresponding region of the brain in relation to the motor homunculus. In our results, subtle differences in measured signal were seen when alternate extremities were utilized which may be a function of the sensor orientation in relation to the activated cortex.

The signals measured correlated with specific limbs in multiple subjects (motor movement, motor imagery, and emotional imagery). After determining the exact location of the broadcasted EMF, these signals could potentially be translated into a functional image of the human brain that could be interpreted by researchers and physicians. In an effort to better understand the ability to interpret the EMF generated by emotional and motor imagery, future studies may investigate baseline data from a large population of subjects as each subject likely has unique characteristics.

Limitations

This study is limited by the relatively small sample size and the novelty of the sensors which limits comparison. Larger sample sizes may be utilized to further assess the reproducibility between subjects. Further studies may also be completed to correlate measured activity with findings of magnetoencephalography to evaluate if there is overlap within any of the recordings. This may allow for visual mapping of activity in the future. With a larger sample size, it may also be possible to identify similarities among patients that may ultimately improve diagnostic utility and could be translated for clinical purposes.

## Conclusions

The intrinsic EMF generated by the human brain can be measured in a non-contact, non-invasive, continuous manner through the skull and scalp using a shielded helmet, as demonstrated in prior studies. It now appears possible to identify changes within that EMF during emotional and motor imagery experimentation in human subjects. Emotional and motor imagery was demonstrated to differ from rest and stereotyped motor activity. Future studies should evaluate similarities between human subjects to improve potential diagnostic utility.
